# Design and Development of Sublingual Printlets Containing Domperidone Nanocrystals Using 3D Melting Solidification Printing Process (MESO-PP)

**DOI:** 10.3390/pharmaceutics15051459

**Published:** 2023-05-10

**Authors:** Lucía Lopez-Vidal, Alejandro J. Paredes, Santiago Daniel Palma, Juan Pablo Real

**Affiliations:** 1 Unidad de Investigación y Desarrollo en Tecnología Farmacéutica (UNITEFA), CONICET, Haya de la Torre y Medina Allemde, Córdoba X5000HUA, Argentina; l.lopezvidal@unc.edu.ar (L.L.-V.);; 2 Departamento de Ciencias Farmacéuticas, Facultad de Ciencias Químicas, Universidad Nacional de Córdoba, Haya de la torre y Medina Allende, Córdoba X5000HUA, Argentina; 3 School of Pharmacy, Medical Biology Centre, Queen’s University Belfast, 97 Lisburn Road, Belfast BT9 7BL, UK; a.paredes@qub.ac.uk

**Keywords:** MESO-PP 3D printing, 3D printing, nanocrystals, nanotechnology, printlets, domperidone, sublingual

## Abstract

Domperidone (DOM) is a drug commonly used to treat nausea and vomiting, as well as gastrointestinal disorders. However, its low solubility and extensive metabolism pose significant administration challenges. In this study, we aimed to improve DOM solubility and avoid its metabolism by developing nanocrystals (NC) of DOM through a 3D printing technology—melting solidification printing process (MESO-PP)—to be delivered via a solid dosage form (SDF) that can be administered sublingually. We obtained DOM-NCs using the wet milling process and designed an ultra-rapid release ink (composed of PEG 1500, propylene glycol, sodium starch glycolate, croscarmellose sodium, and sodium citrate) for the 3D printing process. The results demonstrated an increase in the saturation solubility of DOM in both water and simulated saliva without any physicochemical changes in the ink as observed by DSC, TGA, DRX, and FT-IR. The combination of nanotechnology and 3D printing technology enabled us to produce a rapidly disintegrating SDF with an improved drug-release profile. This study demonstrates the potential of developing sublingual dosage forms for drugs with low aqueous solubility using nanotechnology and 3D printing technology, providing a feasible solution to the challenges associated with the administration of drugs with low solubility and extensive metabolism in pharmacology.

## 1. Introduction

Domperidone (DOM) is a dopaminergic antagonist drug with central antiemetic and peripheral prokinetic properties. Stimulation of dopaminergic receptors inhibits gastric motility, resulting in various gastrointestinal symptoms, such as postprandial bloating and pain, premature satiety, nausea and vomiting. DOM acts by overriding this dopaminergic effect and thus preventing subsequent gastrointestinal symptoms [1,2]. Unlike other dopaminergic antagonist drugs, DOM does not cause significant side effects in the central nervous system (CNS) as it does not cross the blood–brain barrier, which makes it a safer alternative due to the lower incidence of adverse effects [3].

Since it was first synthesized in 1974, it has been widely used as a short-term antiemetic to prevent stomach problems. However, its formulation and oral administration present a series of difficulties. DOM is a class II drug in the Biopharmaceutical Classification System (BCS); i.e., it has a low aqueous solubility (s = 0.986 mg/L) [4], which translates into low and erratic absorption from the gastrointestinal tract [5]. In turn, it undergoes extensive first-pass metabolism in the intestinal wall and liver [6], with a bioavailability of around 15% when taken on an empty stomach and 24% in the presence of food [7]. In this context, it is necessary to administer the drug in a way that avoids drug metabolism and reduces inter- and intra-individual variability in gastrointestinal absorption due to the patients’ diet.

In this regard, sublingual administration, in which the drug is absorbed and passes directly into the systemic circulation [8], is a promising approach to address this problem. Its easy and painless administration, the almost instantaneous onset of drug action, the superior bioavailability (compared to the oral route) and the possibility of being used in the pediatric population and in people with swallowing problems make this route of administration a promising approach when establishing pharmacotherapy [9]. However, there is a series of factors that affects absorption by this route that must be taken into account during the formulation. Primarily, the pKa of the drug must be greater than 2 in an acid and less than 10 in a base to ensure passage through the oral mucosa and, at the same time, it must be highly soluble in salivary secretion [10]. Dom is a weak base with a pKa of 7.9, causing an increase in the molecule’s solubility as the solution’s pH decreases due to ionization. According to the authors of [11], at pH 6.8 (the normal pH of the salivary medium), the solubility of DOM is 27 times lower than at pH 1.2 (gastrointestinal pH). This and its low solubility makes sublingual administration of the drug difficult.

Taking into account the limitations associated with the low solubility of some drugs, our group has worked extensively to obtain nanocrystals (NCs) by a “top-down” method, successfully achieving the nanometrization of different active pharmaceutical ingredients (API) [12,13,14]. NCs are solid particles of a drug with crystalline characteristics and sizes in the nanometric range, typically between 200 and 500 nm [15]. NCs are surrounded by a layer of stabilizer to prevent aggregation and can be prepared either as suspensions—commonly named nanosuspensions—or as powder, which is obtained by removing the solvent from the nanosuspension. Currently, these systems have become one of the simplest and most effective methods to improve the dissolution behavior and bioavailability of poorly soluble drugs [5]. The decrease in particle size and the consequent increase in the surface area of the system are characteristics that could be beneficial for sublingual administration: they would allow greater solubility and speed of dissolution of the drug in the salivary fluid [16], while the high specific surface area promotes a better interaction of nanoparticles with the mucosal surface compared to the conventional drug, thereby promoting its permeation [17]. However, the incorporation of NCs in solid dosage forms (SDF) requires the implementation of techniques that do not rely on specific flow control or high pressures and while allowing NCs to be loaded on a support that facilitates redispersion without generating significant increases in the resulting system size in the process [18]. NCs exhibit unfavorable flow properties, which precludes their utilization in large-scale industrial equipment. Furthermore, nanoparticles tend to aggregate when exposed to physical processes (such as direct compression), since the proximity of their surfaces and short-range thermodynamic interactions promote particle–particle bonding, leading to an increase in size and a loss of their desirable characteristics [19].

Additive manufacturing methods, such as 3D printing (3DP), are shown to be an innovative and versatile alternative for obtaining oral SDF loaded with nanosystems [20]. This technology, which produces solid objects layer by layer following the instructions of a digital model, presents differential capabilities in comparison to the traditional methods used to obtain SDF. Some advantages of these methods are (a) the ability to create, with the same equipment and without changing assembly lines, solid structures of different shapes and sizes without loss of precision; (b) the ability to combine materials with different physicochemical properties (hydrophobicity/hydrophilicity) which can even be placed, without being mixed, in different layers or surfaces of the structure; and (c) the ability to obtain innovative geometries, difficult to achieve with traditional methods, such as hollow or porous structures [21]. Three-dimensional printing is thus able to customize treatments by adapting doses and release profiles just by modifying the geometry, size or internal structure of the digital model.

In recent years, 3DP technology has been used to create sublingual drug delivery systems with customized doses—and even specific shapes—in order to improve personalization, absorption, or patient comfort [22,23]. In the oral administration of DOM, 3DP has been used, on two occasions, to obtain gastroretentive oral pharmaceutical forms [24,25], which are not effective for a rapid onset of drug action. In both works, the authors used the fused deposition modeling (FDM) 3DP technique. This method is characterized by temperatures that are incompatible with NCs’ loading, since they are generally formulated with stabilizers that melt above 55 °C. 

It should be noted that sublingual absorption is highly dependent on the dissolution of the drug in the salivary environment [14], so delivery of many insoluble drugs is limited by this route. Recently, our work group has used the melt solidification printing process (MESO-PP) method to obtain fast-release oral SDFs loaded with 50% albendazole NCs, significantly improving the dissolution properties of this drug in the stomach. This direct extrusion technique is characterized by the absence of solvents, high temperatures and extreme pressures [26,27,28,29,30]—all of which could negatively affect the NCs and their distinctive characteristics. MESO-PP is so far the only method that has been able to load NCs in a SDF at such concentration. The use of MESO-PP technology can not only provide the customization advantages of 3DP, but it can also create NC-loaded SDFs that increase their saturation solubility and concentration gradient, leading to improved adsorption by passive diffusion across the membrane.

The objective of the present project was to obtain domperidone nanocrystals (DOM-NCs) by wet milling technique and integrate them into MESO-PP inks for 3DP to develop fast disintegrating 3D-printed dosage forms (“printlets”) suitable for sublingual administration.

## 2. Materials and Methods

### 2.1. Materials 

DOM was purchased from Todo Droga^®^ (Córdoba, Argentina). PEG 1500 and propylene glycol (PPG) were purchased from Sigma-Aldrich (St. Louis, MO, USA). Poloxamer 407 (P407) was purchased from BASF (Buenos Aires, Argentina), sodium lauryl sulfate (SLS) and croscaramellose sodium (CCS) were purchased from Parafarm^®^ (Barcelona, Spain), sodium starch glycolate (SSG) and sodium citrate (SC) were purchased from Cicarelli^®^ (Santa Fe, Argentina). 

For the milling process, 0.1 mm Zirmil^®^ yttria-stabilized zirconia beads (Saint-Gobain ZirPro Kölh, Germany) were used. Tuberculin syringes (1 mL) (Bremen) were used to load the different components of the mixture into the 3D printer cartridges and a short 19-gauge metal needle (Lhaura) was used for printing. The water used in the study was Milli-Q ultrapure water with a resistivity of 18.2 MΩ at 25 °C and was passed through a filter with a pore size of 0.22 µm. For the dissolution study, hard gelatin capsules (size 0) were purchased from Todo Droga (Córdoba, Argentina). All other reagents were of analytical grade unless otherwise specified.

### 2.2. Methods

#### 2.2.1. Obtaining Domperidone Nanocrystals

##### Nanomilling

A DOM nanosuspension (DOM-NS) was prepared by microsphere-assisted nanomilling using a laboratory-scale mill, NanoDisp^®^ (Córdoba, Argentina) [28]. The equipment consisted of a chamber where the milling material, zirconium beads of 0.1 mm diameter, and the suspension, formed by the drug and stabilizing agents, were housed. Inside the chamber there was an agitator coupled to a motor that worked at high revolutions, generating the collision between the drug particles, the milling agent, and the chamber walls, breaking the drug into nanometer-sized particles. All batches were prepared as follows: a suspension consisting of 80 mL of ultrapure water with a 75:22:3 ratio of DOM:P407:SLS was placed inside the equipment chamber along with 150 g of the milling agent. The process temperature was set at 15 °C and the agitation was at a speed of 1800 rpm for 2 h. Throughout the process, samples were taken every 30 min to evaluate the change in particle size and polydispersity index (PI).

##### Freeze-Drying

In order to obtain domperidone nanocrystals (DOM-NCs) with the smallest possible particle size after solvent removal, different cryoprotectants were tested. For this, the DOM-NS obtained after nanomilling was divided into three equal parts: different cryoprotectants (mannitol and sucrose at 3% P/V) were added to two parts and one part had no additives. The tree samples were frozen using nitrogen and placed in a freeze dryer (Labconco^®^ Freeze Dry System/Freeze Zone 6, Kansas City, MO, USA) at a temperature of −50 °C and a vacuum of 9 × 10^−3^ mBar. After 48 h, the powder was removed from the containers and weighed to determine its yield. Subsequently, the resulting DOM-NCs were stored in plastic containers, hermetically sealed and protected from humidity.

The yield (*Y*) after freeze-drying was determined using Equation (1):

(1) $$Y\%=\frac{Ws}{Wt\;}\times 100$$

where *Ws* is the total weight of powder obtained after lyophilization and *Wt* is the total weight of DOM, P407, SLS, and cryoprotectant—mannitol or sucrose.

#### 2.2.2. Particle Size and Polydispersity Index

Using the dynamic light scattering (DLS) technique, with a measurement angle of 173°, and a Zetasizer^®^ Nano ZS 90 (Malvern Instruments, Malvern, UK), the DOM-NS obtained after the milling process and the DOM-NCs resulting from the freeze-drying were characterized in terms of the particles’ hydrodynamic diameter (size) and polydispersity index (PI). Prior to measuring, the appropriate concentration was achieved by diluting the NS (1:100) in Milli-Q water and redispersing 10 mg of DOM-NCs powder in 2.5 mL of water. The resulting suspensions were shaken manually for 1 min before measuring, which was carried out in triplicate for each sample.

#### 2.2.3. Nanoparticles’ Stability

The stability of DOM-NS stored at 25 °C and in refrigerated conditions (−5 °C) and DOM-NCs with and without cryoprotectant stored at 25 °C, protected from light and humidity, was evaluated in terms of size and *PI* by DLS to assess any changes in nanoparticle size distribution produced by time and storage. For this purpose, the stability index (*SI*) of each formulation was calculated using Equations (2) and (3). The SI of nanoparticles provides an indication of their stability, facilitating comparison between different formulations. The closer the *SI* is to 1, the more stable the formulation is considered to be.

(2) $$SI_{PS}=\frac{PS_{0d}}{PS_{250d}}$$

Equation (2): *PS*_0*d*_ is the mean value of the particle size of DOM-NS at day 0, and *PS*_250*d*_ is the corresponding mean value of DOM-NCs (with and without cryoprotectant) at day 250;

(3) $$SI_{PI}\frac{PI_{0d}}{PI_{250d}}\;$$

Equation (3), *PI*_0*d*_ is the *PI* value of DOM-NC at day 0, and *PI*_250*d*_ is the corresponding value of DOM-NS at day 250. The closer the value of *SI* is to 1, the more stable the NS obtained.

#### 2.2.4. Physical Mixture

A physical mixture (PM) of DOM, P407, and SLS was used as a control for DOM-NCs in the different tests. This was prepared in an agate mortar using the same ratio used for the DOM-NCs (75:22:3, DOM:P407:SLS).

#### 2.2.5. Saturated Solubility

Samples of DOM-NCs, pure drug, and PM were subjected to a solubility saturation assay. For this, an excess of each sample was added separately to 5 mL of simulated salivary medium (pH = 6.8). The samples were incubated for 72 h at 25 ± 0.1 °C in a thermostated bath with continuous agitation at 20 rpm. The obtained samples were filtered through a 0.45 μm membrane filter (Burlington, MA, USA). All samples were prepared in triplicate.

The obtained solutions were adequately diluted and analyzed by UV–vis spectrophotometry at 284 nm (Agilent Cary 60 spectrophotometer) and the percentage of drug dissolved in each medium was determined using calibration curves determined in water (R^2^ = 0.9916) and simulated salivary medium (R^2^ = 0.9932), as appropriate.

#### 2.2.6. NC-Loaded Ink Formulation and Control Inks

The preparation of the NC-loaded ink (DOM-NC-3D) was always carried out as follows: the excipients (PEG 1500, PPG, SLS, SC and SSG) that made up the ink were weighed, added to a beaker and heated at 45 °C. Once melted, the DOM-NCs were gradually incorporated at 45 °C while stirring at 150 rpm ± 10 rpm until the final proportion of 10% (corresponding to 7.5% of pure drug) was reached and it was constantly agitated during 1 h. The printing cartridges (1 mL tuberculin syringes), were then filled with the homogeneous mixture and allowed to stand for 24 h for subsequent printing and characterization. The preparation of control inks (1-DOM-3D and 2-DOM-3D ink) was carried out following the same procedure, except for the addition of DOM-NCs.

#### 2.2.7. Printlet Setup

Using a design platform (www.onshape.com accessed on 8 December 2022), the 3D geometric design of the printlets was created. The design in STL format was loaded into the Repetier Host software (Repetier-Host V 2.1.3), which was used to cut the design into layers, create the internal structure of the printlets and generate printer instruction code (g-code) to carry out the printing process. The final pharmaceutical form then consisted of an internal honeycomb lattice and a fill density of 40%, with no solid top or bottom layer.

#### 2.2.8. Printing Process

The DOM-NCs-loaded printlets (DOM-NC-3D) and the control printlets (1-DOM-3D and 2-DOM-3D) were obtained by the MESO-PP technique [26]. The printing process consisted of using heat to melt the ink contained in the cartridges and extrude it through the printer nozzle (3-Donor^®^ developed by Life SI) while forming the three-dimensional object previously designed following the g-codes. The printing parameters were as follows: printhead temperature 36 °C, platform temperature 25 °C, amount of material per dot 10 nL, printing pressure 0.02 bar, layer printing delay 1200 ms, layer thickness 0.55 mm. The printing speed was 4.5 mm/s, which resulted in a total printing time of 2.54 min per printlet.

#### 2.2.9. Printing Efficiency

The printing efficiency (*PE*)—a variable we defined to evaluate the process according to the formulated ink—was determined using Equation (4). The higher the *PE*, the better the printing process.

(4) $$PE\%=\frac{SP}{PS}\times 100$$

Equation (4): *SP* is the total number of successful impressions obtained and *PS* is the total number of total impressions attempted. A failed impression may be due to a total blockage of the printing system, a partial blockage (which generates gaps and usually uncontrolled flow) or deformations in the desired geometry.

#### 2.2.10. Determination of the pH of the Printlets

The pH of the obtained DOM-NC-3D was determined by dissolving one printlet in 10 mL of distilled water. Once completely dissolved, the pH was measured by immersing the electrode in the resulting solution using a pH meter (Phmetro Adwa Ad1030, Mettler Toledo, OH, USA). The procedure was performed in triplicate.

#### 2.2.11. Weight/Volume Ratio and Weight Variation

According to USP, no more than 2 of the individual masses should deviate from the average mass by more than 5% [30]. To assess the feasibility of dose adjustment by changing the printlet size, the weight/volume ratio was calculated by producing printlets ranging from 100 to 60% of the original size. Each of the resulting printlets were weighed separately and the arithmetic mean and relative standard deviation were calculated. Dimensions were measured with a 0–150 mm Vernier caliper (BTA tools).

#### 2.2.12. Microscopy

##### Scanning Electron Microscopy (SEM) and Energy Dispersive X-ray Spectroscopy (EDS)

To examine the matrix composing the DOM-NCs and the surface morphology and internal structure of the printlets, scanning electron microscopy (SEM) (FEI Inspect 50, USA; high-resolution scanning electron microscope) was carried out at an accelerating voltage of 3–20 kV. Both samples (DOM-NC and DOM-NC-3D) were subjected to an Au coating process before examination. The magnification range varied between 2500× and 50,000×. To determine the distribution of elements in the sample, X-ray energy dispersive spectroscopy (EDS or EDX) was used in conjunction with SEM. ImageJ software (NIH, USA) was used to determine the particle size in the printlets.

##### Hot Stage Microscopy

In order to observe the samples’ behavior at different temperatures, hot stage microscopy (OLYMPUS Model BX-51) was used to observe DOM, DOM-NCs, the DOM-NC-3D, (1- and 2-DOM-3D ink), and the base ink made up of PEG 1500, propylene glycol, CCS, SSG, and SC. Images of the phase transition during the heating process were taken by camera under polarized light for subsequent analysis using INFINITY software. For this purpose, the samples were placed on open glass slides (without coverslips, for better imaging), fixed on the hot stage and heated from 25 to 75 °C at a heating rate of 10 °C/min. Pictures were taken at different times for further analysis and comparison.

#### 2.2.13. Infrared Spectroscopy

Fourier-transform infrared spectra (FTIR) of the samples (DOM-NCs, SLS, P407, DOM, DOM-NC-3D, PEG 1500, SC, CCS, and SSG) were obtained. For this purpose, they were prepared in KBr (100 mg per 2 mg of sample) and scanned from 4000 to 650 cm^−1^ with a resolution of 1 cm^−1^ using a spectrophotometer (FTIR, Agilent Technologies Cary 630, Santa Clara, CA, USA). The data were analyzed with OMNIC^®^ software (Version 8.3.103, Thermo Fisher Scientific, Waltham, MA, USA). 

#### 2.2.14. X-ray Diffraction

A PANalytical X’Pert ProVR X-201 powder diffractometer (PANalytical B.V., Almelo, The Netherlands) was used to obtain the X-ray diffraction (XR) patterns of the samples (DOM-NC, P407, SLS, and DOM). For this purpose, the continuous scanning mode was used at a speed of 2°/min. The range was 5–60° at 2θ with a scanning speed of 0.04° 2θ/s. The DOM-NC and each of the components that made up the DOM-NC-3D were analyzed. The data were processed using OMNIC^®^ software (Thermo Fisher Scientific, USA).

#### 2.2.15. Thermal Analysis

Thermal behavior of the obtained printlets and their components was analyzed by differential scanning colorimetry (DSC). For this purpose, the raw materials used to obtain DOM-NC-3D, DOM-NC-3D, PM, and 1-DOM-3D and 2-DOM-3D inks were subjected to the differential scanning calorimeter (TA Instruments, New Castle, DE, USA). Four milligrams of the samples were weighed and placed in non-airtight aluminum capsules. Measurements were performed in a temperature range from 0 to 260 °C, under a dynamic atmosphere of N_2_ (50 mL/min) and with a heating rate of 10 °C/min. Pre-calibration was performed with indium according to the manufacturer’s recommended protocol. 

For thermogravimetric analysis (TGA), another 4 mg of the samples was placed in aluminum capsules and subjected to a N_2_ flow of 50 mL/min; heating ramp of 10 °C/min in a temperature range of 25 to 250 °C. Pre-calibration of the equipment (TA Instruments, New Castle, DE, USA, Discovery model) of the temperature scale was performed with nickel according to the manufacturer’s recommended protocol. 

In both cases, TRIOS software (Version number 5.1.1, USA) was used for data processing.

#### 2.2.16. Disintegration Test

The disintegration time of the DOM-NC-3D was determined according to Ph. Eur. 5.4 Ed. using a disintegration tester (Tablet Disintegration tester BJ-2, USA). The time required for the total disintegration of 6 prints in 500 mL at 37 ± 0.5 °C of simulated salivary medium was recorded.

#### 2.2.17. In Vitro Dissolution Test 

Dissolution assays of DOM-NC-3D and 1-DOM-3D and 2-DOM-3D printlets were carried out using the USP II drug dissolution apparatus (paddle type) (SOTAX AT 7 Smart, Westborough, MA, USA) in 500 mL of simulated salivary medium (pH = 6.8) at 37 ± 0.1 °C stirred at 50 rpm. Each printlet was accurately weighed before being placed in individual dissolution vessels. Three milliliters of samples were collected from each medium using 0.22 μm filters at different intervals (1, 3, 5, 7.5, 10, 15, 20, and 30 min). Drug content was determined by UV–vis spectrophotometry (Agilent Cary 60 spectrophotometer) at 284 nm. The dissolution tests were performed in triplicate and the results are presented as mean values obtained as a function of time. The dissolution profiles were compared using the similarity factor *f2* (Equation (5)) and the difference factor f1 (Equation (6)).

(5) $$f2=50\times log\left\{{\left(1+\frac{1}{n}\times \overset{n}{\underset{t=1}{\sum }}{\left(Rt-Tt\right)}^{2}\right)}^{0.5}\times 100\right\}$$

Equation (5): Similarity factor *f*2, where *Rt* and *Tt* are the averages of the dissolved percentages of the drug in the reference and the sample at each time interval, respectively.

(6) $$f1=\left\{\left(\overset{n}{\underset{t=1}{\sum }}\left(R^{\prime }t-T^{\prime }t\right)/\overset{n}{\underset{t=1}{\sum }}R\prime \right)\right\}$$

Equation (6): Difference factor *f*1, where *R′t* and *T′t* are the averages of the cumulative dissolved percentages of the drug in the reference and sample, respectively.

#### 2.2.18. Statistical Analysis

The results are expressed as mean values ± standard error of the mean (SD). The software used for statistical analysis was InfoStat (InfoStat Software, Version number 2020, Argentina). An ANOVA test was used for comparison/statistical analysis. Differences were considered statistically significant with a *p* less than 0.05.

## 3. Results and Discussion

### 3.1. Domperidone Nanocrystals

#### 3.1.1. Media Milling and Freeze-Drying

In this study, we employed the microsphere-assisted nanomilling technique to successfully obtain nanocrystals (NC) of domperidone (DOM). This top-down approach allows for easy scalability, enabling the production of large batches of NC while avoiding the use of organic solvents. Other authors have also investigated various methods for producing DOM-NC, including supercritical antisolvent precipitation and a combination of solvent evaporation and sonication [31,32]. Although these techniques have shown potential in improving the properties of DOM, they may not be as practical for industrial-scale production due to various limitations. In contrast, the microsphere-assisted nanomilling technique offers a more practical and efficient approach for large-scale production of DOM-NC. 

Our study demonstrates the feasibility and potential of this technique for industrial-scale production, which could have significant implications for the pharmaceutical industry. When working on obtaining drug NCs, the value of the polydispersity index (PI) is almost as important as the particle size obtained. If the PI obtained is greater than 0.3, the system is considered heterogeneous, indicating that the particle size distribution is variable, which affects solubility and usually results in erratic bioavailability [5]. In our case, we observed an acceptable PI (0.147) after 30 min of the milling process.

The selection of the stabilizing agent is a key step in the process as it should promote the reduction of the particle size of the solid and provide physical stability. To be effective, it should cause wetting of the surface of the solid crystals and provide them with an ionic or steric barrier. In the absence of an appropriate stabilizing agent, the high surface energy of the nanosized particles will cause their aggregation [33]. Previous studies have shown that the combination of an electrostatic and steric stabilizer, also called “electrosteric stabilization”, is more effective in stabilizing drug NCs [34]. In order to generate both steric and electrostatic repulsion, P407 (non-ionic, steric in nature) and SLS (ionic-anionic surfactant) were selected as stabilizers at 22 and 3% *w*/*w*, respectively. Both are solids at room temperature, soluble in water, nontoxic, and recognized as GRAS (generally recognized as safe) products by the US Food and Drug Administration (FDA) [30].

The milling process lasted 2 h and samples were taken every 30 min to observe the change in size and PI (Table 1). After 30 min of grinding, a decrease in particle size from 11,980 nm ± 132 to 248.8 ± 1.4 was observed, indicating that the selected nanometrization method, media milling, efficiently produced DOM-NS.

To find the most favorable conditions for drying, the NS was divided into three equal parts: one was frozen as it was and different cryoprotectants were tested on the others. The three systems were subjected to a freeze-drying process to remove the solvent and finally obtain the solid NCs in powder form (NC 1, 2 and 3). The resulting particle size and PI values are shown in Table 2. The results allowed us to discard sucrose as a cryoprotectant (NC 3), and NC 1 and 2 (without cryoprotectant and with mannitol, respectively) were selected to assess size and PI stability over time and then choose the final formulation.

#### 3.1.2. Storage Stability

**Figure 1:**
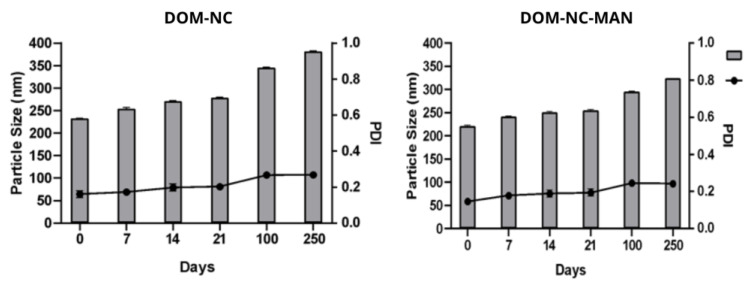
Size and PI values of freeze-dried DOM-NC with and without mannitol for 250 days.

**Figure 2:**
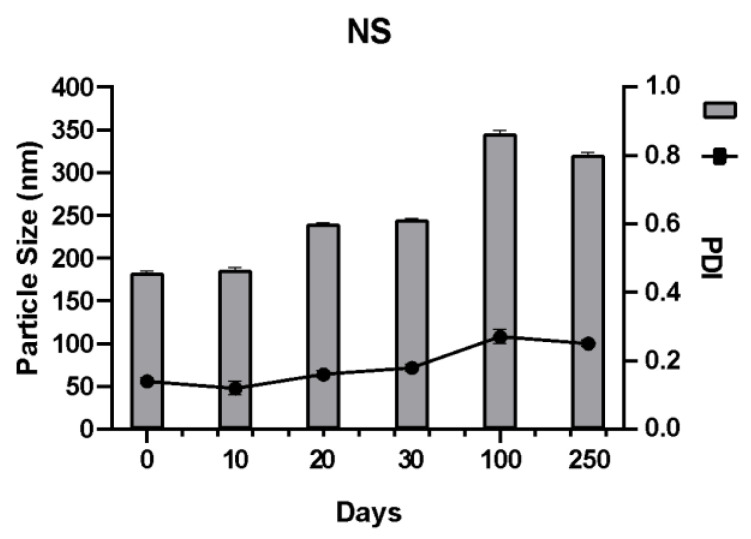
Size and PI values of the NS obtained over 250 days.

An increase in the size of the nanoparticles over time due to agglomeration or crystalline growth (known as Ostwald ripening [35]) would affect the properties of the system, which is why the stability of the DOM-NCs, the NC with mannitol (DOM-NC-MAN) (Figure 1), and the elaborated NS (Figure 2) was evaluated over days in terms of size and PI.

In the case of the NS, it was observed that after 250 days in storage, the particle size and PI remained within acceptable values (320 ± 1.2 nm and a PI < 0.3), the SI_PS_ was 0.57, and the SI_PI_ 0.56. This indicates that drying is not necessary as soon as the milling process is finished, since it is possible to store the DOM-NS. 

In turn, after 250 days, the SI of the NCs was calculated. The DOM-NCs had an SI_PS_ of 0.68 and an SI_PI_ of 0.59, while the DOM-NC-MAN had an SI_PS_ of 0.61 and an SI_PI_ of 0.60. Since the values obtained did not differ significantly, and both formulations remained within acceptable limits after 250 days, DOM-NCs were selected as the final formulation for further work.

#### 3.1.3. Solid State Characterization

##### Infrared Spectroscopy

**Figure 3:**
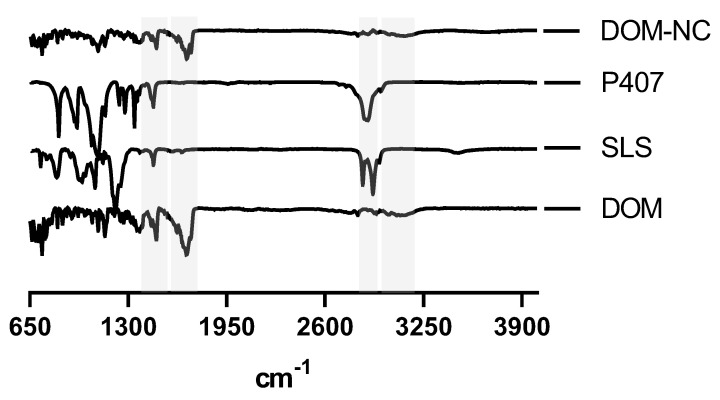
FT-IR analysis of DOM-NC and raw materials.

To study possible intermolecular interactions between the excipients and the drug produced by the milling process, the raw materials and NCs were subjected to FT-IR analysis (Figure 3). The DOM spectrum showed characteristic peaks at 3100, 1692, 1485, and 1383 cm^−1^ corresponding to N-H, C=O, C-N, and two C-O stretching vibrational bands, respectively [36], which were also identified in the spectrum corresponding to the DOM-NCs. P407 and DOM share most of the characteristic peaks of the poloxamer; however, it was possible to identify a peak at 2880 cm^−1^ attributable to the stretching vibration of CH_2_ [37] that was not observed in the spectrum of the drug but could be seen in that obtained from the NCs. It was not possible to identify the presence of characteristic SLS peaks in the NCs obtained, possibly due to the low proportion of this compound in the final product (3%). Finally, no changes or broadening of bands or the presence of new peaks were observed for the NCs obtained, indicating the absence of a chemical interaction between the drug and the excipients used.

##### X-ray Analysis

**Figure 4:**
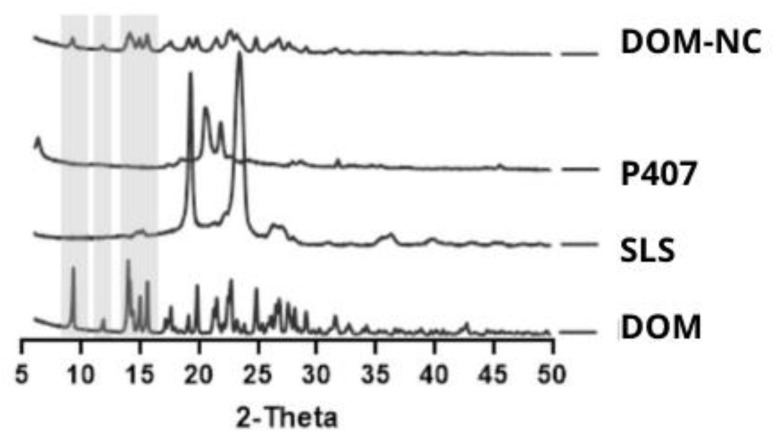
X-ray diffraction patterns of excipients SLS, P407, pure DOM, and DOM-NCs.

It is known that the milling process can generate changes in the crystalline state of the components, such as amorphization or the appearance of polymorphs, phenomena that are often attributed to the high mechanical energy input during the process [38,39]. In this regard, the characteristic peaks of DOM (9, 12 and peaks comprised in the interval between 13 and 16 2θ degrees) were maintained in the Rx pattern of DOM-NC (Figure 4). 

##### Differential Scanning Calorimetry and Thermogravimetric Analysis

**Figure 5:**
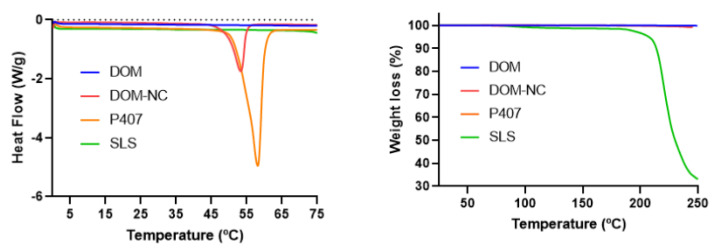
(**left**) DSC and (**right**) TGA of DOM-NCs, raw materials, and PM.

Since the final objective was to obtain printed SDF and MESO-PP uses temperatures in the range of 25–70 °C, it was necessary to evaluate the thermal stability of the DOM-NCs. DOM-NCs, raw materials, and the PM of the components were assessed with DSC and TGA. In the TGA it was possible to observe a loss <0.5% in all formulations between 25 and 70 °C; SLS presents a significant mass loss above 200 °C (Figure 5 left). 

In turn, the DSC thermogram of DOM was characterized by an endothermic peak at 251.54 °C (DHf = 111.94 J/g) and of P407 at 56.25 °C (DHf = 137.27 J/g) in agreement with that reported in the literature [26]. However, in DOM-NC and PM, the peak appeared slightly shifted towards lower temperatures at 53.88 °C (DHf = 38.557 J/g) and 53.13 °C (DHF = 12.339 J/g), respectively, possibly due to an interaction of the poloxamer with the other components (Figure 5 right), something that we had already observed in a previous work with poloxamer 188 [13]. With these results, it was observed that the NCs were optimal to be subjected to the 3DP MESO-PP process.

##### Scanning Electron Microscopy

**Figure 6:**
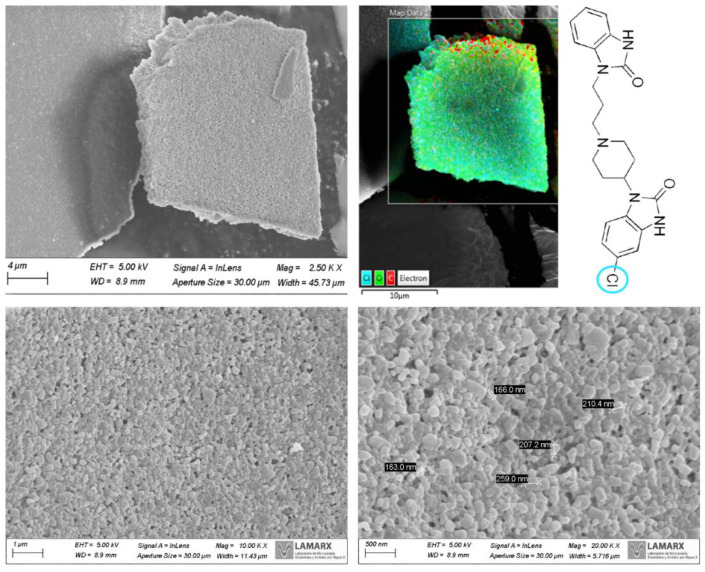
Images taken with scanning electron microscopy of the DOM-NCs.

The DOM-NCs obtained after the freeze-drying process were observed under the microscope, where it was possible to observe the homogeneity of the obtained sample and to measure the particle size (Figure 6).

As it can be seen in the image, the NCs had a particle size of approximately 200 nm, which correlates with the data reported by DLS. To observe drug distribution throughout the samples, an elemental analysis was performed by EDS looking for the chemical element Cl, which is present in the API and is not found in the structure of the rest of the excipients (P407 and SLS). It was observed that the drug was homogeneously distributed throughout the resulting powder.

#### 3.1.4. Solubility Test

DOM is a Class II drug in the BCS, resulting in erratic bioavailabilities and large inter- and intra-individual variability. As shown in Table 3, the solubility of pure drug, nanocrystals (DOM-NC), and physical mixture of the components (PM), showed statistically significant differences in both water and simulated saliva (*p* < 0.05).

The PM increased the solubility of the drug in water and salivary medium 2 and 5 times, respectively, probably due to a decrease in surface tension caused by the surfactant present in the mixture.

On the other hand, nanometrization of the drug produced an increase in solubility with respect to its intrinsic solubility of approximately 4 times in water and 16 times in simulated saliva. This is explained by the Ostwald–Freundlich equation (Equation (7)), where an increase in the specific surface area translates into an increase in the saturation concentration.

(7) $$log\;\frac{Cs}{C\infty }=\frac{2\sigma V}{2.303RT\rho r}\;$$

Equation (7) *Cs*, saturation solubility; *C∞*, solubility of the drug formed by large particles; σ, interfacial tension substance; *V*, molar volume; *R*, gas constant; *T*, absolute temperature; *ρ*, drug density; and *r*, drug particle radius.

### 3.2. Ultra-Fast Ink Release

#### 3.2.1. Ink Formulation

An ultra-rapid release ink was designed with the objective of achieving the disintegration of the printlet as soon as possible. For this purpose, PEG 1500 was used as a carrier due to its low melting point (44 °C) and propylene glycol as a plasticizer to facilitate the printing process and allow the ink to flow easily through the nozzle. In turn, croscaramellose sodium (CCS) and sodium starch glycolate (SSG)—excipients normally used as disintegrants in SDF—and sodium citrate (SC)—generally used in the formulation of effervescent tablets [40]—were used.

Two control inks were also prepared: 1 DOM-3D ink and 2 DOM-3D ink, which were formulated at 46 °C following the proportions indicated in Table 4.

#### 3.2.2. Ink pH

In sublingual formulations, it is important that the pH is in the range between 6.7 and 7, to avoid irritation of the oral mucosa. For this reason, the pH of the elaborated ink was measured to be 6.9 ± 0.1.

#### 3.2.3. Solid State Characterization

##### Hot Stage Microscopy

**Figure 7:**
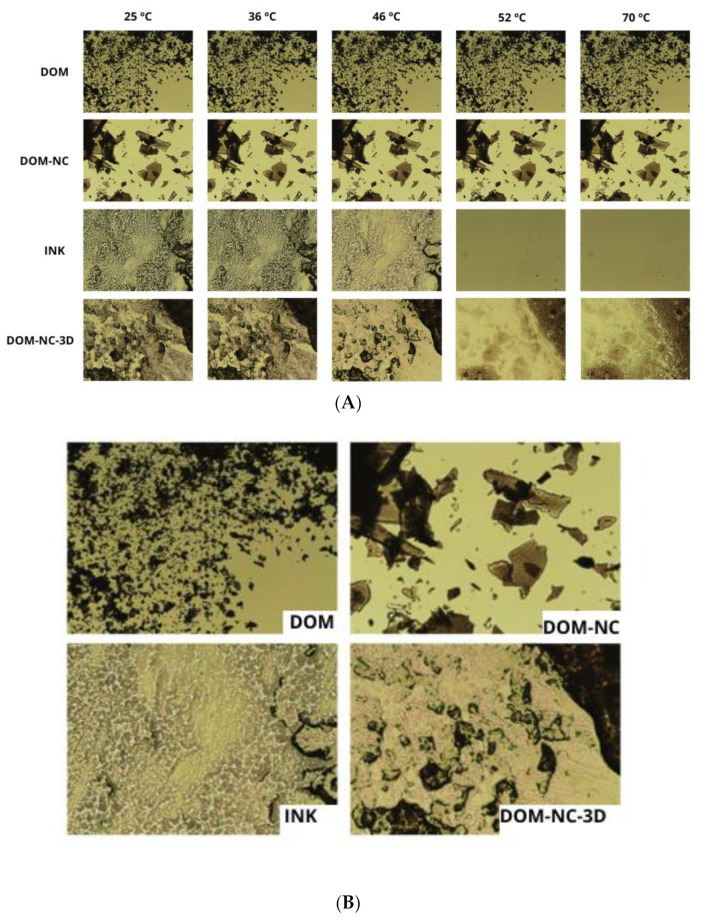
(**A**) images taken with optical microscopy with hot bed at different temperatures; (**B**) images taken by optical microscopy with hot bed at 46 °C (formulation temperature). the magnification used was 10×.

In order to visualize the behavior of DOM-NCs when incorporated in the ink and to observe whether they dissolved or remain suspended in the ink, hot-bed optical microscopy was performed and images were taken at different temperatures in a range from 25 to 75 °C. For this purpose, DOM, DOM-NCs, DOM-NC-3D, 1 and ink 2 DOM-3D inks, and white ink (consisting of PEG 1500, propylene glycol, CCS, SSG, and SC) were observed under the microscope (Figure 7).

As shown in Figure 7A, DOM and DOM-NC maintained their structures unchanged throughout the observed temperature range. In the case of DOM-NCs, we expected to observe a change at 52 °C due to the melting of the poloxamer; however, this could not be seen, possibly due to the low proportion of the poloxamer in the formulation (<25%). On the other hand, it can be observed that the ink began to melt at 46 °C—the formulation temperature. Once the temperature reached 52 °C, no visible solid particles were observed in the mixture, indicating the complete melting of the mixture. As can be seen in more detail in Figure 7B, DOM-NCs did not dissolve in the ink and they remained suspended in the ink.

##### Differential Scanning Calorimetry and Thermogravimetric Analysis

**Figure 8:**
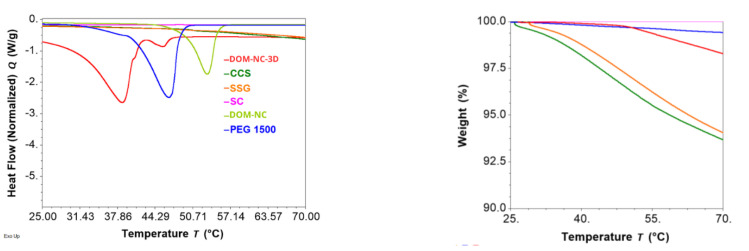
(**left**) DSC and (**right**) TGA of DOM-NC-3D and raw materials.

The TGA of DOM-NC-3D and its components (SSG, CS, CCS, PEG 1500, and DOM-NCs) (Figure 8 right) showed that, in the temperature range used during the printlets production process (ink preparation and 3DP), sodium citrate and PEG 1500 did not present mass losses. In turn, sodium croscaramellose and sodium starch glycolate presented a mass loss of 2.89 and 2.19%, respectively, at 46 °C—the temperature used at the time of ink formulation. The mass loss in the case of DOM-NC-3D at 46 °C was 0.2%, attributable to the CCS and SSG present in the ink. DOM-NCs did not present mass loss in the temperature range studied.

The DSC thermograms (Figure 8 left) show a pronounced endothermic melting peak at 46.64 °C (DHf = 168.19 J/g) for PEG 1500, which is consistent with the reported melting point of this polymer [41]. In turn, a shift towards lower temperatures of the peaks present in the DOM-NC-3D with respect to the pure materials is observed, from 46.64 to 38.69 °C for PEG1500 and from 53.88 to 45.63 °C, respectively, for the peak corresponding to the melting of the poloxamer present in the DOM-NCs. This could be due to the presence of propylene glycol in the ink, which generates a decrease in Tg associated with the plasticizing effect granted [38].

**Figure 9:**
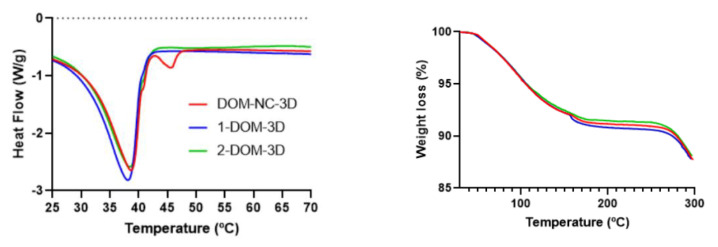
(**left**) DSC and (**right**) TGA of the DOM-NC-3D and the control inks (1-DOM-3D and 2-DOM-3D).

Finally, the DSC thermogram for DOM-NC-3D was compared with the two control inks—1-DOM-3D and 2-DOM-3D (Figure 9 left). It was observed that there was also a shift of the PEG 1500 endothermic peak for the loaded ink. On the other hand, there was no melting peak for the P407 present in 2-DOM-3D ink despite its presence in the formulation—while the melting peak for P407 could be observed at 45.63 °C (DHf = 3.5269 J/g) in the case of DOM-NC-3D. This may be due to a solubilization of the poloxamer in the previously melted PEG1500, an indication of the physical bonding between the drug and the stabilizer resulting from the nanometrization process. 

According to the TGA presented in Figure 9 right, the DOM-NC-3D, 1 DOM-3D ink and 2 DOM-3D ink remained stable under the temperature conditions employed in the MESO-PP method. This stability is crucial to ensure that the inks maintain their desired properties throughout the 3DPprocess. Additionally, the mass loss observed at these temperatures was less than 2%, indicating good thermal stability, which is important for proper storage and handling of the inks. While other authors have used various 3DP methods to obtain sublingual delivery forms [42], it was observed that mass loss could exceed 10% at the higher temperatures used in other 3D printing methods, such as fused deposition modeling (FDM). This suggests that the properties of the ink and the nanocrystals containd within it could be compromised during this type of 3DP process. Moreover, the stabilizer used to obtain the NCs (Poloxamer 407) has a low melting point (55 °C), and exposure to high temperatures would cause it to melt, leading to destabilization of the nanocrystals.

Thermal analysis is relevant in 3DP methods based on hot extrusion, such as MESO-PP. If the printing temperature is not adequate, the viscosity of the ink may be too low, causing the shape to collapse, or too high, preventing the extrusion through the nozzle. Based on the inks’ melting peaks, the printing temperature was defined at 36 °C for both DOM-NC-3D and control inks 1 and 2, ensuring that the material did not melt completely. 

##### FT-IR Analysis

**Figure 10:**
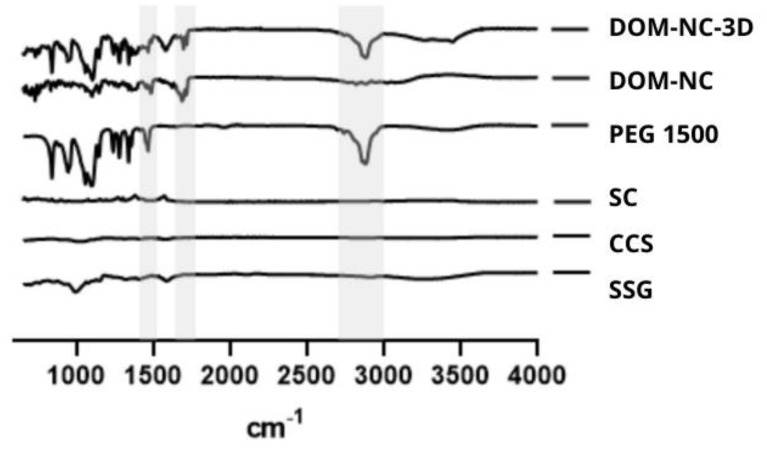
FT-IR analysis of DOM-NC-3D and raw materials.

FT-IR analysis was used to rule out possible chemical interactions between the DOM-NCs and the raw materials used to prepare the DOM-NC-3D. As shown in Figure 8, the characteristic peaks of the DOM-NCs could be clearly identified in the spectrum corresponding to the DOM-NC-3D. The peak corresponding to the C-H vibration in PEG 1500 can be observed in the formulated ink. All observed peaks were attributable to the components of the formulation, indicating that there were no interactions between the excipients and the drug or that they were not strong enough to show these changes (Figure 10).

### 3.3. DOM-NC 3D Printlets

#### 3.3.1. Design of the Dosage Form

**Figure 11:**
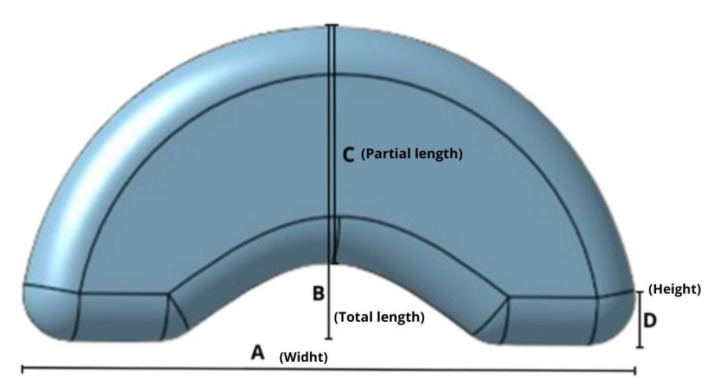
Geometry selected for printing the SDF.

To determine the optimal design for the pharmaceutical form, a study was conducted involving five participants. Two possible geometries were created for the study: the first was an oblong shape measuring 18 mm long by 10 mm wide and 3 mm high, while the second was an oblong shape with a concave section that could be adapted to fit under the sublingual cavity, measuring 20 mm long, 10 mm wide, and 3 mm high. Both designs were intended for a 25 mg dose of DOM. Both shapes were printed using a fused deposition modeling printer (Hellbot^®^, Magna 2 230) with PLA filament and distributed to the participants to place under their tongues. All five individuals chose shape 2 (Figure 11).

Based on these results, the final dosage form was developed based on shape 2, with no top or bottom solid layer. The fill density was determined to optimize solvent ingress and decrease the disintegration time of the print.

#### 3.3.2. Printing of the SDF

The printing process was carried out by placing syringes loaded with ink into the electrically heated alloy tube at the optimal printing temperature (42 °C). Then, the printer’s vertical tower moved while a piston pressed the syringe plunger to gradually deposit the mixture in a semi-solid state onto the print bed, where it solidified at room temperature. This process was repeated layer by layer until the designed three-dimensional shape, previously designed in a computer-aided program, was rendered.

The printing efficiency (PE) was 80%, which means that 13 printing attempts were needed to obtain 10 printlets. The average weight of the obtained printlets was 343.5 ± 19.63 mg, corresponding to a DOM dose of 25 ± 0.2 mg.

**Figure 12:**
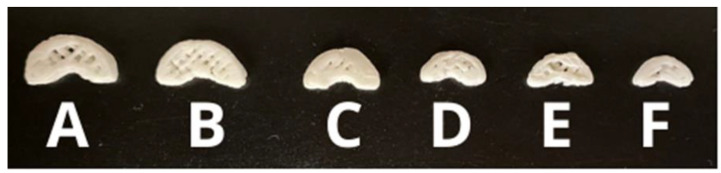
Printed with DOM-NC-3D of different sizes, A being the largest and F the smallest.

Using the previously selected geometry as a reference size (Figure 11), printlets of various sizes were produced (Figure 12). After printing, they were weighed to calculate the theoretical drug dose and their dimensions were measured to calculate the volume (Table 5). It was observed that the printlets had a correlation between volume and average weight of 99% (R^2^ = 0.9888).

Other authors have worked on the use of meloxicam NC for sublingual administration, successfully obtaining fast-disintegrating films using the solvent casting method with ethanol [43]. In a work by Gamal M. Zayed and collaborators, mucoadhesive buccal films of DOM were produced. There was a clear improvement in the drug dissolution profile and a rapid disintegration of the film [44]. However, the drug loading in the film was limited to 10%, as DOM precipitated in the film above this value. In this sense, one of the advantages provided by 3DP in obtaining these SDFs for sublingual administration is the possibility of individualizing pharmacotherapy, since the SDF containing the specific required dose can be printed based on patient data. On the other hand, MESO-PP provides us with the advantage of not requiring the use of organic solvents and allowing NC loading in the system of up to 50% [13]. 

#### 3.3.3. Solid-State Characterization

##### Scanning Electron Microscopy

**Figure 13:**
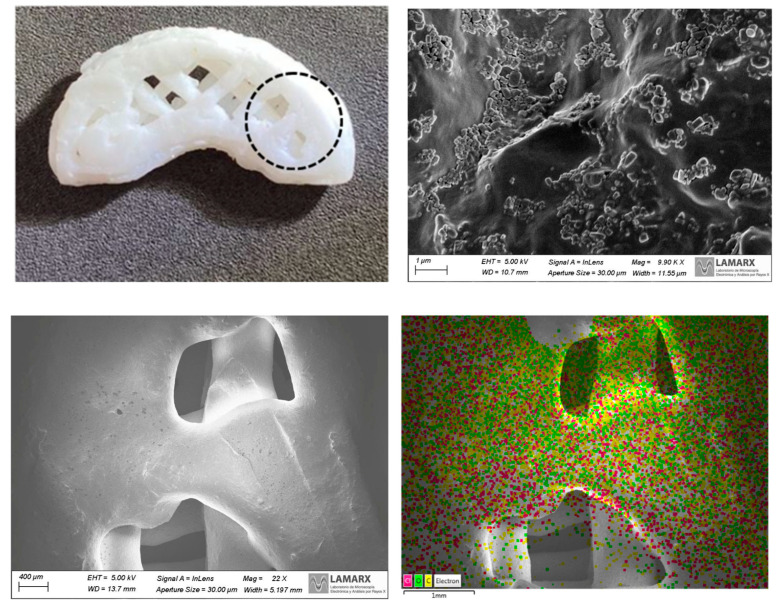
Images taken with scanning electron microscopy of DOM-NC-3D prints. In the figure, the area where the focus was placed in the image to perform the EDS is circled.

Pictures of the DOM-NCs-loaded printlets were used to observe their internal structure and the precision of the mesh that made up the structure (Figure 13). Elemental analysis was also performed by EDS looking for the chemical element Cl, which was present in the API, but not in the rest of the excipients that made up the ink.

As it can be seen in the images, the filaments forming the print were sharp, demonstrating the printer’s ability to accurately obtain a mesh structure. At 9.90 K X magnification, the NCs on the surface of the filaments can also be seen, which could be another indication that no interaction was generated between the DOM-NCs and the excipients. Finally, it can also be observed that Cl was well-distributed along the printlet (3.72%) (fuchsia dots on the image). In the elemental analysis by EDS it can be seen how the chemical element Cl, indicative of the DOM active principle, was homogeneously distributed along the printlet (3.72%), which demonstrates the capacity of the method to obtain printlets with a correct distribution of the drug throughout the SDF.

### 3.4. Disintegration Test

The USP establishes a disintegration time less than or equal to 2 min for pharmaceutical forms for sublingual administration. Thus, an in vitro assay was carried out. The disintegration time of the 6 printed formulations was in the range of 85–90 s, which could be attributed to the high hydrophilicity of the excipients. This result demonstrates the rapid dissolution of the printlet and the rapid release of the drug content, in accordance with the pharmacopoeia for SDF for sublingual administration [30].

### 3.5. Dissolution Study

**Figure 14:**
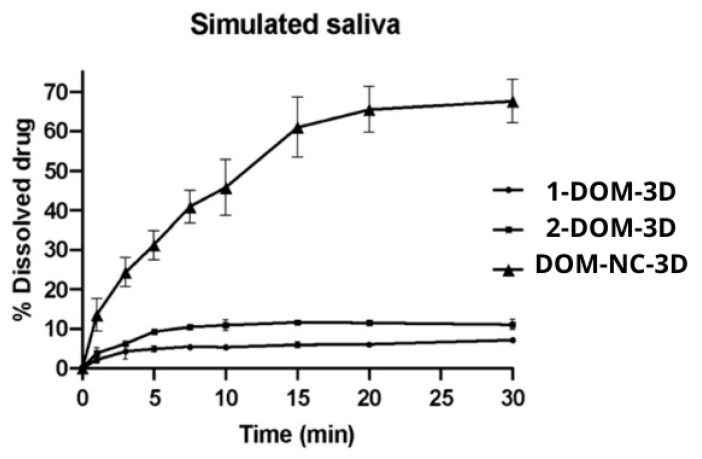
In vitro dissolution profiles of the ink containing DOM-NC and the control inks.

To demonstrate the advantages of NCs, a dissolution test was performed. Printlets of the same shape and size, made with different inks—DOM-NC-3D, 1 DOM-3D, and 2 DOM-3D—were tested. The in vitro dissolution profiles of DOM are shown in Figure 14.

As can be seen, the dissolution profiles of the control inks (1 DOM-3D and 2 DOM-3D) were similar and showed no significant differences (*f*2 = 65.72). This shows that the increase in the solubility of the DOM-NC-3D was not due to the addition of P407 and SLS to the formulation, but rather to the milling process.

After 5 min from the start of the trial, the ink containing NCs had released more than 30% of the drug, while 1 DOM-3D ink had released less than 5% and 2 DOM-3D ink, below 10%. After 30 min, the release of the DOM-NC-3D was close to 70%, while the controls did not exceed 12%. The comparison between DOM-NC-3D and 1 DOM-3D ink resulted in a factor *f*1 = 88.2 and a factor *f*2 = 17.21, while the comparison between DOM-NC-3D and 2 DOM-3D ink resulted in *f*1 = 78.65 and *f*2 = 19.5. A value of *f*1 ≥ 30 and an *f*2 ≤ 50 indicate difference between the curves.

As can be seen in the Noyes–Whitney equation (Equation (5)), the dissolution rate of a drug is conditioned, among other factors, by the surface area available for dissolution and the saturation concentration. The process of particle nanosizing results in a decrease in particle radius with a consequent increase in saturation concentration according to the Ostwald–Freundlich equation (Equation (4)). At the same time, this size reduction leads to an increase in the exposed surface area, which increases the surface area available to come into contact with the dissolution medium, thereby accelerating the dissolution process. The combination of both phenomena could explain the differences observed in Figure 14.

(8) $$\frac{dC}{dt}=\frac{DS}{h}\left(Cs-\frac{Xd}{V}\right)$$

Equation (8) *dC*/*dt,* dissolution rate; *D*, diffusion coefficient; *S*, surface area; *h*, diffusion distance; *Cs*, saturation solubility; and *Xd/V,* concentration around the particles.

## 4. Conclusions

The selected top-down technique proved to be an efficient method for the nanometrization of DOM. The NCs obtained in this work showed an increase in drug solubility, along with a stability of at least 250 days and no observable physicochemical changes at any stage of the manufacturing process. On the other hand, the MESO-PP 3D printing proved to be efficient to uniformly load the DOM-NCs in the printlets and to produce SDF that can be administered sublingually with rapid disintegration and an improved drug dissolution profile. These results demonstrate the potential of these technologies to be used together as a way to improve the low solubility and extensive metabolism of DOM, while also allowing customization of size and dose according to the needs of patients. 

## Figures and Tables

**Table 1:** Particle size and polydispersity index (PI) throughout the milling process.

Time (min)	Z-Average (nm)	PI
0	11,980 ± 132	0.83
30	248.8 ± 1.4	0.147
60	200.6 ± 2.0	0.149
90	185.8 ± 0.7	0.156
120	183.2 ± 0.9	0.14

**Table 2:** Particle size and polydispersity index (PI) of redispersion of lyophilized NCs with different cryoprotectants.

NC	Cryoprotector	Particle Size (nm)	PI	Performance (%)
1	-	233.3 ± 0.6	0.161 ± 0.01	93.55
2	Mannitol (3% P/V)	221.2 ± 1.8	0.145 ± 0.01	90.2
3	Sucrose (3% P/V)	222.5 ± 1.8	0.225 ± 0.01	87.9

**Table 3:** Values obtained from the solubility test for DOM, the PM of DOM, P407, and SLS and DOM-NCs in water and simulated saliva.

Medium	DOM	PM	DOM-NCs
Water	0.008 ± 0.002 mg/mL	0.015 ± 0.007 mg/mL	0.031 ± 0.003 mg/mL
Simulated Saliva	0.0026 ± 0.0006 mg/mL	0.014 ± 0.001 mg/mL	0.042 ± 0.003 mg/mL

**Table 4:** Final percentage composition of the inks produced.

Components	Proportion of DOM-NC-3D Components (%)	Components	Proportion of 1 DOM-3D Ink Components (%)	Proportion of 2 DOM-3D Ink Components (%)
CCS	5	CCS	5	5
SSG	5	SSG	5	5
SC	3	SC	3	3
PEG 1500	70	PEG 1500	70	70
Propylene glycol	7	Propylene glycol	7	7
(DOM-NCs) DOM	7.5	DOM	10	7.5
P407	2.4	P407	0	2.4
SLS	0.1	SLS	0	0.1

**Table 5:** Weights and dimensions of the different sizes of printed matter produced, where A is the length, B the upper radius, C the lower radius, and D the height of the printed matter.

Form	Weight (mg)	Dimensions (mm)				Volume (mm^3^)	DOM (mg)
		A	B	C	D		
A	343.5	19.65	9.6	7.65	2.9	824.2	25.7
B	322.8	18.05	9.4	7.1	2.9	724.3	24.2
C	266.8	15.9	9	6.5	2.75	563.8	20
D	167.3	13.5	7.3	5.4	2.6	372.8	12.5
E	132.5	12.4	6.6	5.3	2.4	295.6	9.9
F	101.3	11.7	6.35	5	1.75	194.1	7.6

## Data Availability

The data presented in this study are available on request from the corresponding author.
